# Translational research in type 2 diabetes

**DOI:** 10.1186/1479-5876-10-S2-A21

**Published:** 2012-10-17

**Authors:** Guang Ning

**Affiliations:** 1 Ruijin Hospital affiliated Shanghai JiaoTong University School of Medicine, Shanghai, China

## 

The risk factors for type 2 diabetes, and the association between diabetes and cancer are two hot topics in translational endocrinology that have caused researchers’ high attention. Bisphenol A (BPA) is one of the world’s highest production volume chemical in use. People widespreadly and continuously expose to BPA, and whether BPA associates with human health needs further research. The association between diabetes and cancer has been realized as a very important issue in the field of diabetes and diabetes care. However, how diabetes is linked with cancer and whether the duration of diabetes, as well as the degree of glycemic controls modify the risk of cancer are not well defined.

We recruited 3423 participants aged 40 years or older from Songnan Community, Baoshan District, Shanghai, and all the participants were underwent a 75-gram oral glucose tolerance test and blood and urine samples were collected. Risk evaluation of cancers in Chinese diabetic Individuals: a longitudinal (REACTION) study is an effort to evaluate thoroughly the risk of site specific cancers in Chinese diabetic individuals. In 2011, a study population of 250,000 people was identified from 25 communities across China, aims to research the interaction between type 2 diabetes and cancer, as phase I of a prospective study.

Our study showed that the detection rate of urinary BPA was 87.7%, with a median value of 0.81 ng/mL. Higher levels of urinary BPA associated with a higher prevalence of type 2 diabetes (OR:1.37, 95%CI: 1.08-1.74), generalized obesity(OR: 1.50, 95%CI: 1.15-1.97), abdominal obesity(OR:1.28, 95%CI: 1.03-1.60), insulin resistance(OR: 1.37, 95%CI: 1.06-1.77), and low-grade albuminuria (OR: 1.23, 95%CI: 1.13-1.34). Urinary BPA was positively associated with free triiodothyronine (FT3) (men: β = 0.011, P = 0.003; women: β = 0.013, P < 0.001) and negatively associated with thyroid-stimulating hormone (TSH) (men: β = -0.065, P = 0.002; women: β = -0.11, P < 0.001), and higher urinary BPA was associated with high thyroid function (OR: 1.68, 95% CI: 1.24-2.27). Results from the REACTION study indicated that type 2 diabetes associated with higher prevalence of all types of cancer and digestive system malignant tumors.

BPA might be a risk factor for endocrine diseases, and people should eliminate BPA exposure in daily life. The REACTION study will provide clinical evidence for the interaction between diabetes and cancers.

